# Associations of high-altitude polycythemia with polymorphisms in *PIK3CD* and *COL4A3* in Tibetan populations

**DOI:** 10.1186/s40246-018-0169-z

**Published:** 2018-07-27

**Authors:** Xiaowei Fan, Lifeng Ma, Zhiying Zhang, Yi Li, Meng Hao, Zhipeng Zhao, Yiduo Zhao, Fang Liu, Lijun Liu, Xingguang Luo, Peng Cai, Yansong Li, Longli Kang

**Affiliations:** 1 Key Laboratory for Molecular Genetic Mechanisms and Intervention Research on High Altitude Disease of Tibet Autonomous Region, School of Medicine, Xizang Minzu University, Xianyang, 712082 Shaanxi China; 2 Key Laboratory of High Altitude Environment and Genes Related to Diseases of Tibet Autonomous Region, School of Medicine, Xizang Minzu University, Xianyang, 712082 Shaanxi China; 3 Ministry of Education Key Laboratory of Contemporary Anthropology, Collaborative Innovation Center for Genetics and Development, School of Life Sciences, Fudan University, Shanghai, 200433 China; 4 Division of Human Genetics, Department of Psychiatry, Yale University School of Medicine, New Haven, CT 06510 USA; 5 Six Industrial Research Institute, Fudan University, Shanghai, 200433 China

**Keywords:** High-altitude polycythemia, PIK3CD, COL4A3

## Abstract

**Background:**

High-altitude polycythemia (HAPC) is a chronic high-altitude disease that can lead to an increase in the production of red blood cells in the people who live in the plateau, a hypoxia environment, for a long time. The most frequent symptoms of HAPC include headache, dizziness, breathlessness, sleep disorders, and dilation of veins. Although chronic hypoxia is the main cause of HAPC, the fundamental pathophysiologic process and related molecular mechanisms responsible for its development remain largely unclear yet.

**Aim/methods:**

This study aimed to explore the related hereditary factors of HAPC in the Chinese Han and Tibetan populations. A total of 140 patients (70 Han and 70 Tibetan) with HAPC and 60 healthy control subjects (30 Han and 30 Tibetan) were recruited for a case-control association study. To explore the genetic basis of HAPC, we investigated the association between HAPC and both phosphatidylinositol-4,5-bisphosphonate 3-kinase, catalytic subunit delta gene (*PIK3CD*) and collagen type IV α3 chain gene (*COL4A3*) in Chinese Han and Tibetan populations.

**Results/conclusion:**

Using the unconditional logistic regression analysis and the false discovery rate (FDR) calculation, we found that eight SNPs in *PIK3CD* and one SNP in *COL4A3* were associated with HAPC in the Tibetan population. However, in the Han population, we did not find any significant association. Our study suggested that polymorphisms in the *PIK3CD* and *COL4A3* were correlated with susceptibility to HAPC in the Tibetan population.

## Introduction

High-altitude polycythemia (HAPC) is a chronic high-altitude disease, characterized by excessive erythrocytosis. The clinical HAPC is diagnosed by a hemoglobin concentration ≥ 19 g/dL for females and ≥ 21 g/dL for males, according to the criteria established in the VI World Congress on Mountain Medicine and High-altitude Physiology in 2004 [1]. More than 140 million people are living at high altitudes above 2500 m worldwide, majorly in the Andes, Ethiopian Highlands, and Qinghai-Tibet Plateau [2]. The Qinghai-Tibet Plateau is the highest plateau in the world, which covers a large area with low oxygen in natural environment, and millions of people are living and working in this region. It is well known that the body’s hemoglobin concentration increases due to the hypoxic environment of high altitude, and therefore, this response is crucial for people who adapt to live at high altitudes. Some studies show that a number of populations suffer from chronic mountain sickness because they stay long at high altitudes [3]. HAPC mainly leads to a significant increase in blood viscosity, causing damage to microcirculatory and immune response disturbances such as vascular thrombosis, extensive organ damage, and sleep disorders [4, 5]. It is reported that the prevalence of HAPC in the Qinghai-Tibet Plateau is around 5 to 18% [1], and the prevalence of HAPC increases with the altitude. As the construction of the Qinghai-Tibet Railway has been completed, a number of Han populations migrate to Tibet. The incidence of HAPC among immigrants is significantly higher than the high-altitude natives [6]. As the Tibetan population keeps genetic adaptations, they can easily adapt to the high-altitude hypoxia environment, for example, showing lower hemoglobin levels and lower hematocrit. Many studies have noted that there are some significant differences in the genomes between immigrants and high-altitude natives, which indicates that genetic factors may contribute to the development of HAPC, although the molecular mechanisms and pathogenesis are still under study. In our study, we aimed to investigate the associations between susceptibility to HAPC and two new candidate genes that are related to the oxygen metabolism in red blood cells but have not been reported before.

The first candidate, *PIK3CD*, encodes the p110δ catalytic subunit of phosphoinositide 3-kinaseδ (PI3Kδ), a member of a big family of metalloenzymes. PI3Kδ is a heterodimer comprising the p110δ and p85 family regulatory subunit and expressed predominantly in leukocytes. Therefore, it plays an important role in the proliferation, survival, and activation of leukocytes [7–9]. The expression pattern and functions of *PIK3CD* are very important in PI3K/Akt pathway. Recently, research studies revealed that PI3K/Akt mediated the stabilization of HIF-1α (hypoxia-inducible factors-1α) [10], and it was also involved in the increase of HIF-1α protein level [11]. Meanwhile, HIF-1α plays an important role in transcriptionally upregulating erythropoietin (EPO) in hypoxia and affecting the amount of red blood cells [12].

The second candidate, *COL4A3*, encodes a subunit of type IV collagen that is a structural protein of the alveolar extracellular matrix (ECM) and mostly found in the kidney, lung, and basement membranes. It is located at 2q35-q3 and mainly contains 51 exons [13]. Type IV collagen is involved in various physiological conditions, including aging, diabetes, kidney disease, scarring, and pulmonary fibrosis [14]. The ECM is important to the structure and function of cell types. It contributes to many processes, such as cellular proliferation, differentiation, migration, and apoptosis [15].

## Results

**Fig. 1:**
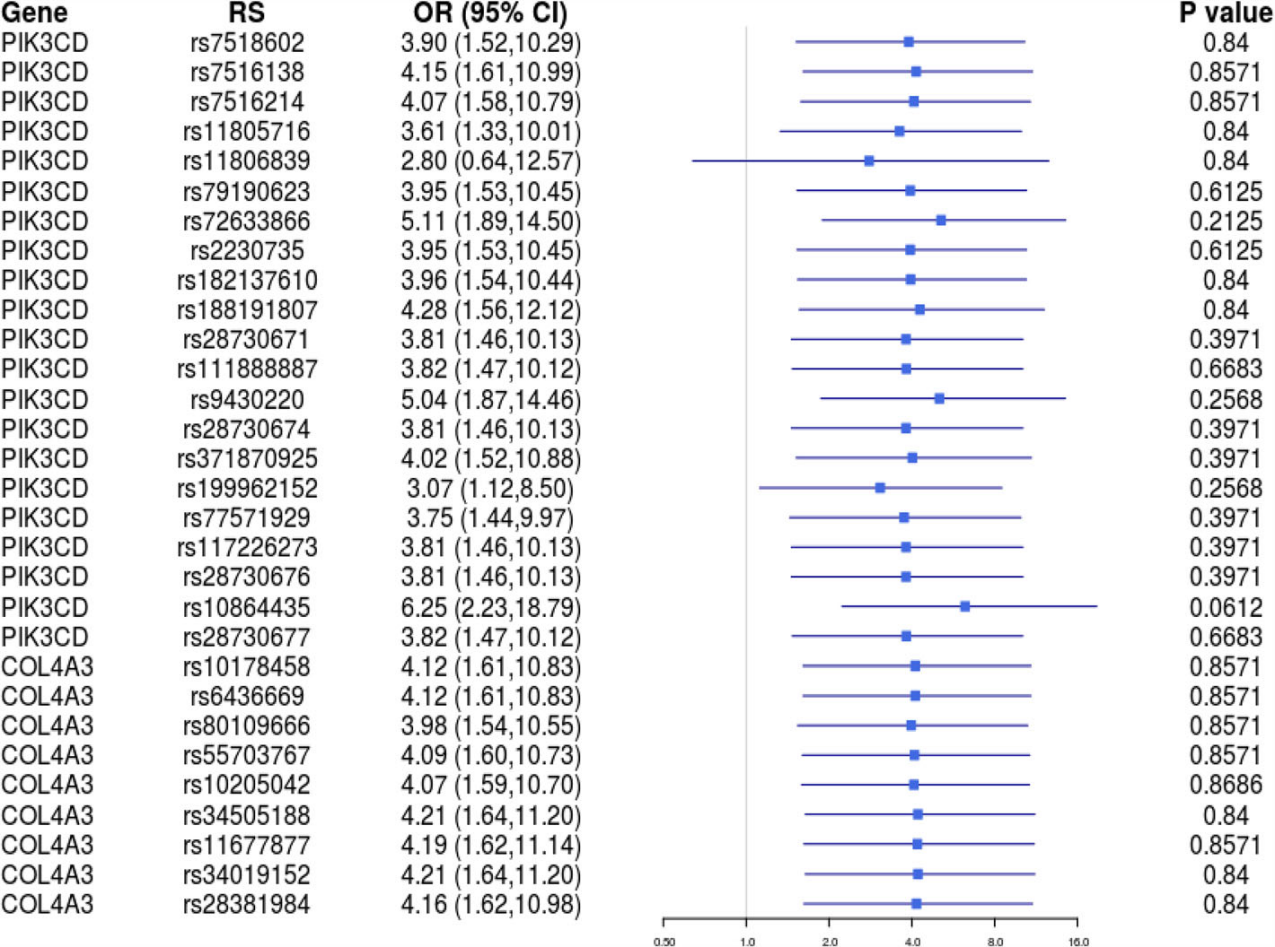
Forest plots for the ORs in the Han population

**Fig. 2:**
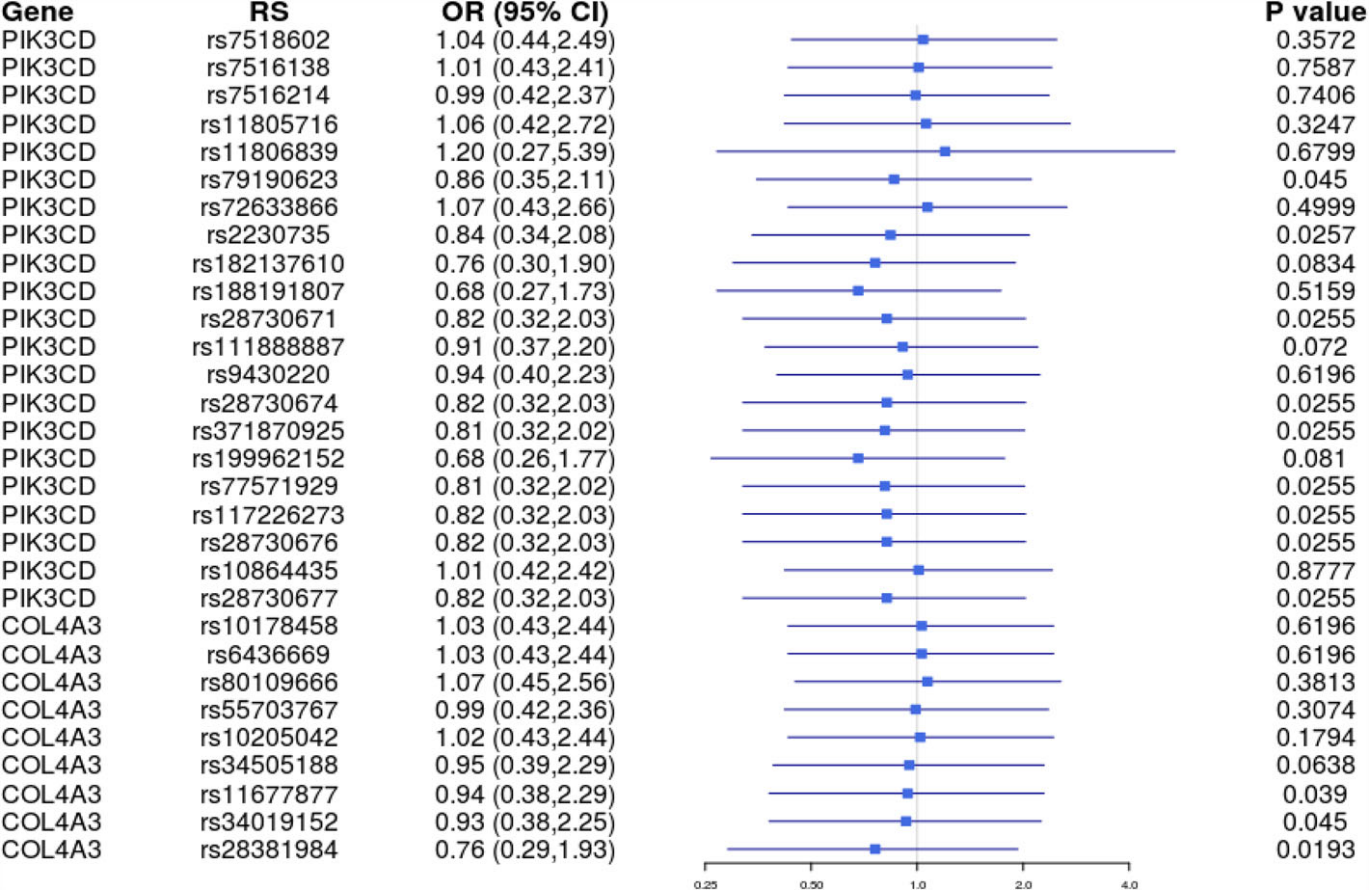
Forest plots for the ORs in the Tibetan population

The demographics of HAPC patients and controls are shown in Table 1. The basic characteristics of candidate SNPs in the Han and Tibetan subjects are summarized in Table 2 (Fig. 1) and Table 3 (Fig. 2). We analyzed the associations between SNPs and HAPC using unconditional logistic regression analysis. In the Han population, rs72633866 (*P*1 = 0.033 before adjustment and *P*2 = 0.014 after adjustment for age), rs9430220 (*P*1 = 0.081 and *P*2 = 0.029), rs199962152 (*P*1 = 0.024 and *P*2 = 0.034), and rs10864435 (*P*1 = 0.013 and *P*2 = 0.002) in *PIK3CD* were significantly associated with HAPC. In the Tibetan subjects, rs2230735 (*P*1 = 0.008 and *P*2 = 0.008), rs28730671 (*P*1 = 0.007 and *P*2 = 0.007), rs111888887 (*P*1 = 0.034 and *P*2 = 0.034), rs28730674 (*P*1 = 0.007 and *P*2 = 0.007), rs371870925 (*P*1 = 0.007 and *P*2 = 0.007), rs199962152 (*P*1 = 0.045 and *P*2 = 0.040), rs77571929 (*P*1 = 0.005 and *P*2 = 0.005), rs117226273 (*P*1 = 0.007 and *P*2 = 0.007), rs28730676 (*P*1 = 0.007 and *P*2 = 0.007), and rs28730677 (*P*1 = 0.007 and *P*2 = 0.007) in *PIK3CD* were significantly associated with HAPC. Furthermore, rs34505188 (*P*1 = 0.028 and *P*2 = 0.028), rs11677877 (*P*1 = 0.013 and *P*2 = 0.013), rs34019152 (*P*1 = 0.018 and *P*2 = 0.018), and rs28381984 (*P*1 = 0.001 and *P*2 = 0.001) in *COL4A3* were associated with HAPC.

**Table 1:** Demographics of the control individuals and patients with high-altitude polycythemia

Variables	Han		Tibetan	
	Case (*n* = 70)	Control (*n* = 30)	Case (*n* = 70)	Control (*n* = 30)
Male	35	15	35	15
Female	35	15	35	15

**Table 2:** Basic information of candidate SNPs in Han subjects

SNP_ID	Gene	Alleles A/B	Case (*N*)			Control (*N*)			OR (95% CI)	*P*	*P*1	*P*2
			AA	AB	BB	AA	AB	BB				
rs7518602	PIK3CD	C/T	4	16	50	0	6	23	3.899 (1.515–10.290)	0.961	0.261	0.425
rs7516138	PIK3CD	G/A	4	19	47	0	11	18	4.150 (1.615–10.989)	0.993	0.959	0.800
rs7516214	PIK3CD	G/A	4	19	46	0	11	18	4.070 (1.582–10.786)	0.993	0.924	0.829
rs11805716	PIK3CD	T/C	13	17	32	3	9	14	3.610 (1.335–10.014)	0.961	0.516	0.488
rs11806839	PIK3CD	G/C	7	11	12	2	6	6	2.800 (0.640–12.570)	0.993	0.627	0.616
rs79190623	PIK3CD	C/T	63	7	0	23	6	0	3.952 (1.533–10.448)	0.766	0.160	0.245
rs72633866	PIK3CD	G/A	64	4	0	22	6	0	5.107 (1.892–14.498)	0.236	0.033	0.014
rs2230735	PIK3CD	A/G	63	7	0	23	6	0	3.952 (1.533–10.448)	0.766	0.160	0.245
rs182137610	PIK3CD	A/C	63	7	0	24	5	0	3.961 (1.540–10.444)	0.961	0.321	0.510
rs188191807	PIK3CD	G/A	57	3	0	23	3	0	4.280 (1.564–12.125)	0.961	0.287	0.486
rs28730671	PIK3CD	C/T	65	5	0	23	6	0	3.808 (1.463–10.129)	0.509	0.061	0.129
rs111888887	PIK3CD	T/C	65	5	0	24	5	0	3.816 (1.471–10.117)	0.821	0.141	0.312
rs9430220	PIK3CD	T/C	37	28	4	9	18	2	5.044 (1.871–14.464)	0.245	0.081	0.029
rs28730674	PIK3CD	A/G	65	5	0	23	6	0	3.808 (1.463–10.129)	0.509	0.061	0.129
rs371870925	PIK3CD	T/C	65	3	0	24	5	0	4.016 (1.520–10.884)	0.509	0.050	0.112
rs199962152	PIK3CD	A/G	63	2	0	22	5	0	3.065 (1.118–8.502)	0.245	0.024	0.034
rs77571929	PIK3CD	T/C	64	5	0	23	6	0	3.737 (1.439–9.970)	0.509	0.065	0.132
rs117226273	PIK3CD	G/T	65	5	0	23	6	0	3.808 (1.439–10.129)	0.509	0.061	0.129
rs28730676	PIK3CD	T/C	65	5	0	23	6	0	3.808 (1.436–10.129)	0.509	0.061	0.129
rs10864435	PIK3CD	C/T	63	7	0	20	9	0	6.247 (2.235–18.787)	0.051	0.013	0.002
rs28730677	PIK3CD	G/A	65	5	0	24	5	0	3.816 (1.471–10.117)	0.821	0.141	0.312
rs10178458	COL4A3	T/C	0	9	61	0	4	25	4.116 (1.069–10.826)	0.993	0.900	0.803
rs6436669	COL4A3	A/G	0	9	61	0	4	25	4.116 (1.069–10.826)	0.993	0.900	0.803
rs80109666	COL4A3	G/A	49	19	2	22	7	0	3.984 (1.545–10.552)	0.993	0.433	0.707
rs55703767	COL4A3	G/T	53	15	2	21	7	1	4.088 (1.600–10.732)	0.993	0.732	0.762
rs10205042	COL4A3	C/T	2	15	53	1	7	21	4.073 (1.592–10.704)	0.993	0.732	0.869
rs34505188	COL4A3	G/A	46	19	5	18	11	0	4.214 (1.637–11.199)	0.993	0.787	0.587
rs11677877	COL4A3	A/G	46	19	5	17	12	0	4.187 (1.625–11.138)	0.993	0.997	0.725
rs34019152	COL4A3	G/A	46	19	5	18	11	0	4.214 (1.637–11.199)	0.993	0.787	0.587
rs28381984	COL4A3	C/T	27	32	11	9	16	4	4.160 (1.623–10.984)	0.993	0.711	0.599

*SNP* single-nucleotide polymorphism, *OR* odds ratio, *95% CI* 95% confidence interval, *P value* FDR-calculated *P* value, *P1 P* value calculated by unconditional logistic regression analysis, *P2 P* value adjusted for age

**Table 3:** Basic information of candidate SNPs in Tibetan subjects

SNP_ID	Gene	Alleles A/B	Case (*N*)			Control (*N*)			OR (95% CI)	*P*	*P*1	*P*2
			AA	AB	BB	AA	AB	BB				
rs7518602	PIK3CD	C/T	5	29	36	1	10	19	1.043 (0.438–2.490)	0.612	0.240	0.238
rs7516138	PIK3CD	G/A	11	26	33	3	13	14	1.015 (0.428–2.411)	0.992	0.735	0.733
rs7516214	PIK3CD	G/A	11	26	32	3	13	14	0.992 (0.416–2.366)	0.982	0.688	0.691
rs11805716	PIK3CD	T/C	15	27	17	6	8	13	1.061 (0.416–2.718)	0.555	0.207	0.206
rs11806839	PIK3CD	G/C	5	7	4	2	9	3	1.205 (0.270–5.393)	0.893	0.593	0.612
rs79190623	PIK3CD	C/T	58	10	1	18	11	1	0.862 (0.349–2.106)	0.081	0.018	0.018
rs72633866	PIK3CD	G/A	57	8	1	25	2	0	1.071 (0.434–2.656)	0.791	0.366	0.367
rs2230735	PIK3CD	A/G	59	10	1	17	12	1	0.844 (0.337–2.079)	0.046	0.008	0.008
rs182137610	PIK3CD	A/C	57	10	1	18	9	1	0.764 (0.301–1.897)	0.150	0.050	0.045
rs188191807	PIK3CD	G/A	53	5	1	22	5	0	0.685 (0.266–1.726)	0.791	0.454	0.396
rs28730671	PIK3CD	C/T	63	6	1	19	10	1	0.821 (0.324–2.035)	0.046	0.007	0.007
rs111888887	PIK3CD	T/C	61	6	1	21	8	1	0.906 (0.368–2.205)	0.130	0.034	0.034
rs9430220	PIK3CD	T/C	29	34	5	12	14	4	0.940 (0.395–2.234)	0.851	0.536	0.535
rs28730674	PIK3CD	A/G	63	6	1	19	10	1	0.821 (0.324–2.035)	0.046	0.007	0.007
rs371870925	PIK3CD	T/C	64	4	1	20	9	1	0.812 (0.320–2.017)	0.046	0.007	0.007
rs199962152	PIK3CD	A/G	58	4	1	19	6	1	0.685 (0.256–1.766)	0.146	0.045	0.040
rs77571929	PIK3CD	T/C	63	5	1	19	10	1	0.814 (0.320–2.024)	0.046	0.005	0.005
rs117226273	PIK3CD	G/T	63	6	1	19	10	1	0.821 (0.324–2.035)	0.046	0.007	0.007
rs28730676	PIK3CD	T/C	63	6	1	19	10	1	0.821 (0.324–2.035)	0.046	0.007	0.007
rs10864435	PIK3CD	C/T	55	15	0	25	4	1	1.011 (0.423–2.417)	0.992	0.879	0.878
rs28730677	PIK3CD	G/A	63	6	1	19	10	1	0.821 (0.324–2.035)	0.046	0.007	0.007
rs10178458	COL4A3	T/C	4	21	45	4	7	19	1.026 (0.432–2.442)	0.851	0.539	0.537
rs6436669	COL4A3	A/G	4	21	45	4	7	19	1.026 (0.432–2.442)	0.851	0.539	0.537
rs80109666	COL4A3	G/A	60	9	1	28	2	0	1.070 (0.449–2.559)	0.655	0.271	0.267
rs55703767	COL4A3	G/T	47	21	2	18	8	4	0.990 (0.415–2.360)	0.524	0.184	0.184
rs10205042	COL4A3	C/T	0	23	47	4	8	18	1.017 (0.425–2.437)	0.305	0.102	0.102
rs34505188	COL4A3	G/A	42	20	8	10	14	6	0.948 (0.390–2.292)	0.115	0.028	0.028
rs11677877	COL4A3	A/G	42	21	7	10	13	7	0.941 (0.385–2.290)	0.070	0.013	0.013
rs34019152	COL4A3	G/A	42	20	8	10	13	7	0.928 (0.380–2.254)	0.081	0.018	0.018
rs28381984	COL4A3	C/T	24	39	7	23	6	1	0.761 (0.294–1.928)	0.035	0.001	0.001

The abbreviations were the same as Table 2

**Fig. 3:**
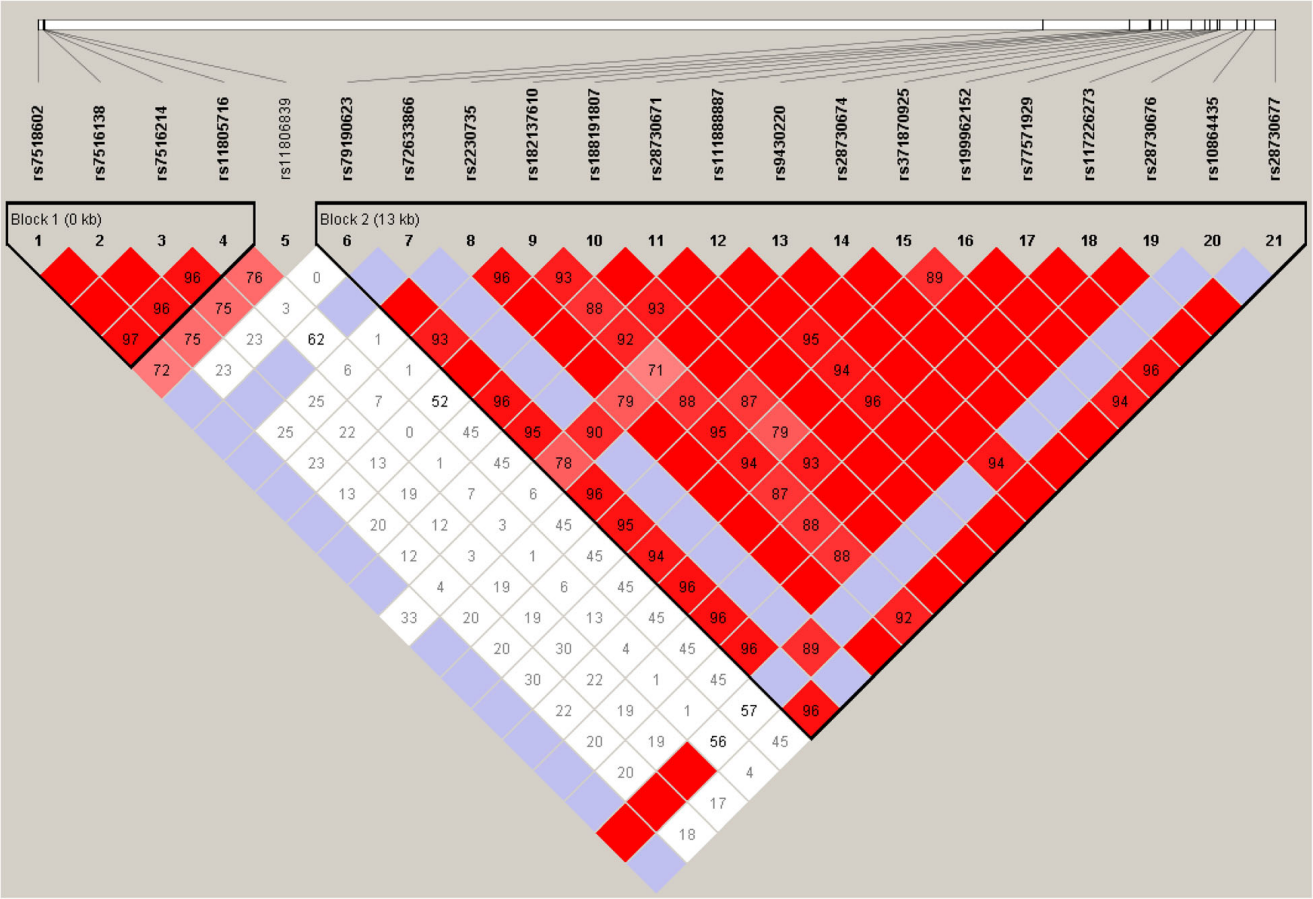
Haplotype block map for the 15 *PIK3CD* SNPs

**Fig. 4:**
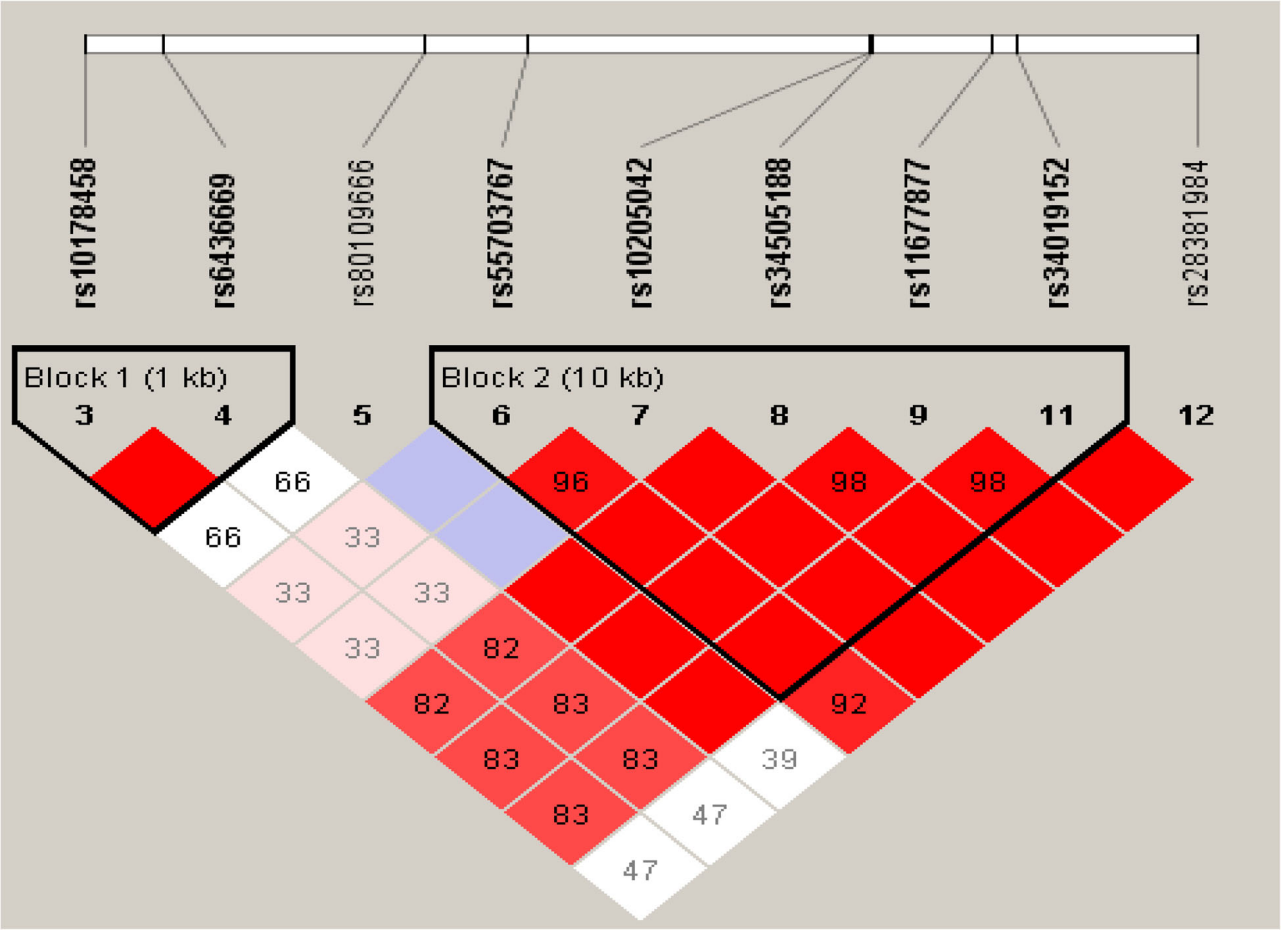
Haplotype block map for the 21 *COL4A3* SNPs

After using FDR to correct for multiple comparisons, in the Tibetan subjects, we found that rs2230735 (OR = 0.844, 95% CI = 0.337–2.079, *P* = 0.046), rs28730671 (OR = 0.821, 95% CI = 0.324–2.035, *P* = 0.046), rs28730674 (OR = 0.821, 95% CI = 0.324–2.035, *P* = 0.046), rs371870925 (OR = 0.812, 95% CI = 0.320–2.017, *P* = 0.046), rs77571929 (OR = 0.814, 95% CI = 0.320–2.024, *P* = 0.046), rs117226273 (OR = 0.821, 95% CI = 0.324–2.035, *P* = 0.046), rs28730676 (OR = 0.821, 95% CI = 0.324–2.035, *P* = 0.046), rs28730676 (OR = 0.821, 95% CI = 0.324–2.035, *P* = 0.046), and rs28730677 (OR = 0.821, 95% CI = 0.324–2.035, *P* = 0.046) in *PIK3CD* were significantly associated with HAPC. Furthermore, rs28381984 (OR = 0.761, 95% CI = 0.294–1.928, *P* = 0.035) in *COL4A3* was associated with HAPC in the Tibetan population. But in the Han population, we did not find any significant association. In addition, using haplotype analysis, two blocks were detected among the *PIK3CD* SNPs (Fig. 3): block 1 contains rs7518602, rs7516138, rs7516214, and rs11805716 and block 2 contains rs79190623, rs72633866, rs2230735, rs182137610, rs188191807, rs28730671, rs111888887, rs9430220, rs28730674, rs371870925, rs199962152, rs77571929, rs117226273, rs28730676, rs10864435, and rs28730677. Two blocks were detected among the *COL4A3* SNPs too (Fig. 4): block 1 contains rs10178458 and rs6436669 and block 2 contains rs55703767, rs10205042, rs34505188, rs11677877, and rs34019152. These SNPs within the same genes showed strong linkage in-between.

## Discussion

Tibet covers a vast area with a harsh hypoxic natural environment. According to a report in 2006, approximately 12 million people permanently settled down in this region. This number constantly increases every year; the increase mainly comes from the Han population that are emigrating from plain areas [16]. HAPC is a serious disease that threatens the health of people in the plateau area, especially those who have emigrated from a low-altitude area. In the past, a large number of patients with HAPC have been investigated with a focus on the pathophysiologic mechanisms of this disease. Nevertheless, a lot of questions remain to be elucidated. With the completion of the human genome project, much research has been shifted to human genetic variation, which was one of NIH’s Roadmap Initiatives for 2008 [17]. As we all know, in Tibet, in order to adapt to altitude hypoxia, the body increases the hemoglobin concentration to increase the efficiency of carrying oxygen, and this response is crucial for the Han population who adapt to live at high altitudes. Compared with the Han people, the Tibetan population keeps genetic adaptations; they can easily adapt to the high-altitude hypoxia environment. Several studies have indicated that natural selection associated with high-altitude adaptation appears to act on genes in the hypoxic response pathway to regulate erythrocyte production, possibly to prevent or reduce erythrocyte growth [18–22]. Recently, significant progress has been made in the study of the genetic basis of HAPC in Tibetans and Han, and some studies have confirmed that many genes are associated with HAPC. Namely, integrin subunit alpha 6 (ITGA6), erb-b2 receptor tyrosine kinase 4 (ERBB4), EPH receptor A2 (EPHA2), angiotensinogen (AGT), and endothelial PAS domain protein 1 (EPAS1) have been reported to play important roles in HAPC in Tibetans and Han [23–26]. Of these genes, EPHA2 can affect erythrocyte production by regulating EPO production and EPAS1 has been implicated as making the greatest contribution to genetic adaptation to high altitude and to the low Hb concentrations observed in the Tibetan population [18, 21]. Some research have shown that genetic variants selected for adaptation at extreme environmental conditions not only increase cancer risk later on age but may also be the downregulation of erythropoiesis in Tibetans in high altitude [22, 27, 28]. In our study, we found rs2230735, rs28730671, rs28730674, rs371870925, rs77571929, rs117226273, rs28730676, rs28730677, and rs28381984 in *PIK3CD* and *COL4A3* were significantly associated with decreased HAPC risk in the Tibetan population. However, in the Han population, we did not find any significant association. Inspired by the development of genome research and the genetic findings of high-altitude natives, we consider that genetic factors may be involved in the formation of such kind of disease. Our study revealed an association of HAPC with SNPs in *PIK3CD* and *COL4A3* in the Tibetan population.

*PIK3CD* gene encodes P110δ catalytic subunit that is expressed predominantly in leukocytes and plays a vital role in the phosphoinositide 3-kinase (PI3K)/Akt signaling pathway. According to recent reports, p110δ contributes to the activation of Akt and cell proliferation in primary AML (acute myeloid leukemia) cells [29, 30]. PI3K signaling contributes to many processes, including cell cycle progression, proliferation and differentiation, survival, and migration [8, 9, 31]. PI3K/Akt pathways are critical to HIF-1α transcriptional activity in hypoxia. HIF-1, which is basically a heterodimer transcription factor, composed of HIF-1α and HIF-1β subunits, serves as a central regulator of metabolic adaptation to low oxygen [32]. The HIF-1α subunit is stabilized under hypoxia, translocating to the nucleus, forming a heterodimer with HIF-1β, and transactivating its target genes including EPO. HIF-1α is a factor that was originally thought to be bound to the 3′ enhancer region of the EPO genes, controlling for 100–200 genes that are involved in angiogenesis, glycolysis, and erythropoiesis [33]. The main organ for EPO production is the liver during the fetus stage, whereas it becomes the kidney after birth. However, there is small amount of expression in the other organs of the body, such as the brain, spleen, lungs, testis, and placenta. Further, it is a necessary glycoprotein, which does not only promote the maturation of red blood cell from erythroid progenitors but also mediates erythropoiesis. It is identified as an inducer of erythropoiesis and can promote excessive cell production. In addition, previous studies showed that inhibited PI3K/Akt signaling pathway led to decreased hematopoietic stem cell (HSC) proliferation. This suggests that such kind of pathway is important for HSC proliferation. HSC is involved in the formation of HAPC, expansion of the population, and enforcement of erythroid lineage-committed differentiation [34]. Therefore, we speculate that PIKCD may affect the generation of EPO and the decrease of HSC appreciation through the PI3K/Akt signaling pathway. In this study, we show here for the first time that the *PIK3CD* gene plays a crucial role in the production of erythrocyte, so *PIK3CD* has a significant influence on the formation of HAPC.

*COL4A3* is an important risk gene for HAPC that is also linked to many diseases such as the Alport syndrome, focal segmental glomerulosclerosis, and type 2 diabetes [35–37]. Furthermore, it is important to the structure and function of various cell types and contributes to a variety of processes. Although the functional effects of the polymorphisms have not yet been elucidated fully, our current results show that the variants may have an effect on *COL4A3* expression or activity. Therefore, it may play an important role in modulating the susceptibility to HAPC. By searching the KEGG pathway database, we found that *COL4A3* can bind to receptors on cell surface and promote the activation of PI3K/Akt. Under hypoxic conditions, this pathway can promote the production of hypoxia-inducible factor and increase the cell cycle, and thereby promote the increase of EPO and the amount of red blood cells. Therefore, it is speculated that *COL4A3* may affect the production of EPO through the PI3K/Akt signaling pathway, thus affecting the production of red blood cells. Consequently, *COL4A3* gene may be a useful marker for the formation of HAPC. We also show here for the first time that the *COL4A3* gene plays a crucial role in the production of erythrocyte. Meanwhile, based on the results of our research, *COL4A3* was significantly associated with erythropoiesis in hypoxia. It is suggested that gene polymorphisms may be relevant to the susceptibility to HAPC.

The genome research era has also opened the road to studying the basis of susceptibility to chronic mountain sickness (CMS) [38]. Gene polymorphisms have set the platform for the analysis of the molecular mechanisms of adaptation to life at high altitudes [39]. Tibet, an average elevation above 4000 m, is commonly regarded as the “Roof of the World” and has a unique genetic background and dietary and lifestyle habits. In this study, we have suggested that several genetic polymorphisms are associated with susceptibility to HAPC and each polymorphism may contribute to only a small relative risk of HAPC. It shows a complex interplay between exposure to hypoxic environmental stimuli and genetic background. There are important discoveries revealed by the studies, but there are still a lot of limitations. Due to these limitations, the study power of this paper is limited. On the other hand, the functions of the genetic variants and their mechanisms have not been evaluated in this study. In a following study, we will use animal models to verify the experimental results, to more clearly illustrate how the two genes affect erythrocytosis, which signaling pathways are involved in the formation of the disease, and to try to elucidate the functions of the genetic variants and mechanisms with HAPC.

## Conclusion

We analyzed SNPs in *PIK3CD* and *COL4A3* and identified a relationship between genetic polymorphisms and HAPC in the Tibetan people. This study sets out to improve the quality of life of people living in the Qinghai-Tibet Plateau, determines paramount insights into the etiology of HAPC, and may provide more guidance for such people with regard to prolonged and healthy living. However, additional genetic risk factors and functional investigations should be identified in order to further confirm our results.

## Materials and methods

### Study populations

A total of 140 patients (70 Han and 70 Tibetan) with HAPC and 60 healthy control subjects (30 Han and 30 Tibetan) were recruited for a case-control association study. The 200 subjects who participated in this research had resided at an altitude of above 4000 m, and these samples were collected from the General Hospital in Tibet Military Region and the second People’s Hospital of Tibet Autonomous Region. Written informed consent was obtained from each individual. Patients met the diagnostic criteria for HAPC, i.e., males with hemoglobin ≥ 21 g/dL or females with hemoglobin ≥ 19 g/dL, and had no high-altitude cerebral edema and chronic respiratory disorders or secondary polycythemia due to hypoxemia caused by certain chronic diseases. Moreover, subjects have no endocrinological, nutritional, and metabolic diseases. Healthy individuals were randomly selected as controls. The experimental protocol was established by the Ethics Committee of the Xizang Minzu University.

### Epidemiological and clinical data

We used a standardized epidemiological questionnaire to collect demographic and clinical data, including information on gender, age, residential region, ethnicity, family history of cancer, and education status. Furthermore, the patient information was collected through physicians or from medical chart review. All participants signed informed consent, and 5 ml of peripheral blood was taken from each participant in this study.

### Selection of SNPs and methods of genotyping

Thirty SNPs from *PIK3CD* and *COL4A3* were chosen for analysis in this study, including 21 SNPs in *PIK3CD* and 9 SNPs in *COL4A3* with minor allele frequency (MAF) > 0.05 in the Asian population HapMap database, and SNP genotyping was performed utilizing Illumina sequencing platform for exon sequencing of PIKCD and COL4A3. Because the genetic background of Han and Tibetan populations has not been compared yet, we selected these two candidate genes based on their relations to the oxygen metabolism in red blood cells, which were related to high-altitude adaptation in the Chinese Han and Tibetan populations.

### Statistical analysis

The data were analyzed using an R program, Haploview, and Excel. Unconditional logistic regression analysis was used to calculate odds ratios (ORs), 95% confidence intervals (CIs), and *P* values for comparisons between cases and controls. Multiple comparisons were corrected using FDR, and FDR-corrected *P* < 0.05 was considered to indicate a significant difference.

## Abbreviations

95%CI: 95% confidence intervals
AGT: Angiotensinogen
AML: Acute myeloid leukemia
CMS: Chronic mountain sickness
COL4A3: Collagen type IV α3 chain gene
ECM: Extracellular matrix
EPAS1: Endothelial PAS domain protein 1
EPHA2: EPH receptor A2
ERBB4: Erb-b2 receptor tyrosine kinase 4
FDR: False discovery rate
HAPC: High-altitude polycythemia
HIF-1α: Hypoxia-inducible factors-1α
HSC: Hematopoietic stem cell
ITGA6: Integrin subunit alpha 6
OR: Odds ratio
PIK3CD: Catalytic subunit delta gene
